# Qi-Long-Tian formula extract alleviates symptoms of acute high-altitude diseases via suppressing the inflammation responses in rat

**DOI:** 10.1186/s12931-021-01645-8

**Published:** 2021-02-12

**Authors:** Xing Fu, Chunyan Yang, Bing Chen, Kexing Zeng, Siyuan Chen, Yi Fu

**Affiliations:** 1 Beijing University of Chinese Medicine, Beijing, 100029 China; 2 Division of Lung Disease, The Third Affiliated Hospital of Yunnan University of Chinese Medicine, Kunming Municipal Hospital of Traditional Chinese Medicine, Kunming, 650500 Yunnan China; 3 Function Teaching and Research Section, School of Medicine, Kunming University, Kunming, 650214 Yunnan China; 4 Yunnan University of Chinese Medicine, Kunming, 650500 Yunnan China; 5 The Third Affiliated Hospital of Yunnan University of Chinese Medicine, Kunming Municipal Hospital of Traditional Chinese Medicine, No. 2628 Xiangyuan Street, Chenggong District, Kunming, 650500 Yunnan China

**Keywords:** Acute hypobaric hypoxia, Qi-Long-Tian, mRNA-seq, Acute high-altitude diseases, Inflammation

## Abstract

**Background:**

Chinese Yunnan Province, located in the Yunnan–Guizhou Plateau, is a famous tourist paradise where acute high-altitude illness common occurs among lowland people visitors due to non-acclimatization to the acute hypobaric hypoxia (AHH) conditions. Traditional Chinese medicine, such as Qi-Long-Tian (QLT) formula, has shown effectiveness and safety in the treatment of acute high-altitude diseases. The aim of this study was to clarify the therapeutic mechanisms of this traditional formula using a rat model in a simulated plateau environment.

**Methods:**

Following testing, lung tissue samples were evaluated by hematoxylin–eosin staining and for biochemical characteristics. mRNA-Seq was used to compare differentially expressed genes in control rats, and in rats exposed to AHH and AHH with QLT treatment.

**Results:**

Inflammation-related effectors induced following QLT treatment for AHH included MMP9 and TIMP1, and involved several phosphorylation signaling pathways implicated in AHH pathogenesis such as PI3K/AKT and MAPK signaling.

**Conclusion:**

This study provides insights into the major signaling pathways induced by AHH and in the protective mechanisms involved in QLT formula activity.

## Background

The northern part of Yunnan Province, China is located on a plateau with an average altitude of over 2000 m. With increasing altitude, the atmospheric pressure decreases exponentially, resulting in a progressive reduction in the ambient partial pressure of oxygen (PO_2_), termed hypobaric hypoxia. When the altitude increases by 100 m, the atmospheric pressure usually drops by 5 mmHg, and the PO_2_ drops by 1 mmHg. Millions of individuals, including the military, mountain rescuers, and mountaineers, currently work or live at high altitudes, and are exposed to the risk of mountain sickness. Physiological studies have shown that the altitude suitable for human survival is between 500 and 2000 m [1]. Individuals experience difficulty breathing and altitude symptoms appear above 2000 m, while saliva secretion begins to decrease at 3000 m. Most individuals experience a state of incapacitation at 6000 m and loss of consciousness and hallucinations occur at 7000 m [2].

When lowland people visit plateaus, a syndrome caused by poor adaptability to the acute hypobaric hypoxia (AHH) environment called acute mountain sickness (AMS) may occur [3]. The principal symptoms of AMS include headache, nausea, vomiting, fatigue, dizziness, and sleep disturbance [4]. Although AMS has been recognized over the past two centuries, little is known about the fundamental causes of these symptoms [5]. Yunnan Province is a tourist paradise, where the number of tourists exceeded 600 million in 2020. It is foreseeable that AMS would seriously threaten the health of travelers visiting the plateau.

A series of physiological responses occur to adapt to AHH conditions. In some cases, these maladaptive responses may predispose affected individuals to various forms of high-altitude illness. The lungs are the first organ to sense changes in barometric pressure and the PO_2_ in the environment and are closely associated with the body's maladaptive responses to AHH [6], which result in significant injuries caused by hypobaric hypoxia, such as high-altitude pulmonary edema (HAPE).

HAPE is noncardiogenic pulmonary edema that may occur in unacclimatized persons within 2 to 4 days of ascent to altitudes above 2500 m [7]. Clinical findings include tachycardia, tachypnea, low-grade fever, hypoxemia, and unilateral or bilateral inspiratory crackles [8]. Risk factors for HAPE include a prior history of the disorder, male sex, rapid ascent, higher altitudes, pre-existing respiratory infection, and intense exercise [9–11]. Susceptible individuals develop exaggerated hypoxic pulmonary vasoconstriction and may experience a large rise in pulmonary artery pressure when exposed to hypoxia. As these changes are distributed unevenly within the pulmonary vascular bed, regional over perfusion of capillaries occurs, leading to a ‘stress failure’ of the blood-gas barrier, increased permeability, and pulmonary edema [12]. This noninflammatory process may be accelerated by impaired alveolar fluid clearance [13].

Traditional Chinese medicine (TCM), which has long been practiced in the clinic in Asian countries, is an important choice for preventing and treating disease. Formulae based on TCM theories are reported to be effective in treating hypoxia pulmonary hypertension which is a symptom of HAPE at the early stage. The Qi-Long-Tian (QLT) formula is composed of three herbs (*Rhodiola, Panax notoginseng*, and dried pheretima at a ratio of 10:5:2) [14]. As a component of QLT, *Rhodiola* has been used in the clinic as a lung protecting agent and can improve cardiopulmonary function, alleviate pulmonary hypertension, and reduce the probability of the occurrence of HAPE [15, 16]. A previous study has shown that this formula reduced endothelin-1, vascular endothelial growth factor, interleukin (IL)-1β, and tumor necrosis factor (TNF)-α levels, and upregulated nitric oxide synthase (NOS) expression in experimental rats with hypoxia pulmonary hypertension [17, 18], but the underlying mechanisms are still unclear.

In the present study, we investigated the mRNA profile of lung tissues from rat models exposed to conditions of normoxia, AHH, or to AHH with Qi-Long-Tian treatment. The results showed that several inflammation-related mediators were significantly induced under AHH conditions, which also responded to QLT treatment. We deduce that these molecule candidates may mediate the action of QLT in vivo. This study provides deeper insight into the mechanisms of action of QLT in the treatment of AHH-related diseases.

## Methods

### Preparation of the extract of QLT formula

The composition of the QLT formula includes *Rhodiola sachalinensis*, *Panax notoginseng*, and dried *Pheretima asiatica* at a ratio of 10:5:2. The raw medicinal materials were obtained and prepared by the Kunming Municipal Hospital of TCMs. The materials were diluted 10 times in distilled water (w/v) and boiled twice for 1 h each time under continuous stirring. After filtration, the residue was boiled with 8 volumes of purified water for another 2 h and filtered. Thereafter, the combined filtrates were subjected to centrifugation at 1500×*g* and the supernatants were concentrated under vacuum to a final concentration of 2 g/mL and stored at − 80 °C until use.

### Animals

Specific pathogen-free male Wistar rats (230 ± 20 g; 6-weeks-old) were obtained from the Laboratory Animal Center of Kunming Medical University. All rats were maintained in an environment at 23 ± 2 °C with 50 ± 5% humidity and a 12 h light–dark cycle. Water and food were provided to animals ad libitum. The experiments started after a week of adaptation. The experimental protocol was approved by the Experimental Animal Care and Ethics Committees of Kunming Municipal Hospital of Traditional Chinese Medicine (Approval No.: R-082018112).

### Rat model preparation and administration

The 130 rats were randomized and divided into the blank (I), AHH (II), and AHH with QLT treatment (III) groups. Animals of the blank group (n = 10) remained at sea-level atmospheric pressure within the same laboratory conditions. Animals of groups II and III (60 rats for each) were exposed to AHH for a total of 24, 48, or 72 h (20 rats for each duration) in a decompression chamber (Feng Lei, Guizhou, China) at a simulated altitude of 6000 m. The animals of the hypobaric hypoxia group III received 8 g/kg (20 rats for each duration) QLT by gavage with once a day for the duration of the experiment. The rats of group II received the same volume of saline intragastrically. The airflow of the hypobaric hypoxia decompression chamber was maintained 10 L/min during exposure to prevent the accumulation of exhaled gases.

### Serum samples

The rats were euthanized with CO_2_ asphyxia for 5 min. Blood samples (4 mL from each individual rat) were collected from the carotid artery into 5% volume heparin tubes (Venoject II, Terumo, Tokyo, Japan) and immediately centrifuged at 3000×*g* for 10 min at 4 °C. The serum was separated and stored at − 80 °C until analysis.

### Hematoxylin–eosin (HE) staining

Paraffin tissue sections were dehydrated under standard protocols, including clearing in xylene (3 times), and dehydration in anhydrous alcohol (95% alcohol, 80% alcohol, 75% alcohol), and distilled water, successively. Paraffin tissue sections were stained with hematoxylin for 5 min, rinsed using tap water, and blotted dry. Next, they were differentiated using ethanolic acid for 30 s, soaked in warm water at 50 °C for 5 min, and drained. Slides were incubated with eosin stain for 2 min and followed by routine dehydration, including 95% alcohol, anhydrous alcohol, xylene carbolic acid, xylene, in order. Finally, slides were sealed with a neutral resin.

### Immunohistochemistry

The tissue specimens were embedded in paraffin and sectioned continuously. The sections were placed in a 65 °C oven overnight, then dewaxing and hydration were carried out. Endogenous peroxidase was blocked by 3% H_2_O_2_. The sections were placed in citrate buffer solution and a microwave heating method was used to repair the antigen. Next, the sections were blocked with 5% normal sheep serum at room temperature (RT) for 20 min. Primary antibody was added at 4 °C overnight. A biotin-conjugated secondary antibody (1:200) was added and incubated at 37 °C for 30 min. HRP-labeled streptavidin solution was added and incubated at 37 °C for 30 min. After DAB staining, the sections were dyed with hematoxylin at RT for 2 min, then dehydrated and sealed with neutral resin. The following antibodies were used: CD3 (1:150, Abcam, ab16669), CD68 (1:50, Abcam, ab955), HIF-1α (1:1000, Abcam, ab8366), VEGF (1:100, Thermo, M808), CGB (1:600, Abcam, ab202972), EPO (1:500, Abcam, ab273070).

### RNA extraction

RNA extraction was carried out as previously described [19]. In brief, 50–100 mg tissue was placed in a 1.5 mL EP tube (RNase free), and 1 mL Trizol was added to homogenize tissues. The tissue was allowed sit for 5 min at RT. Chloroform (0.2 mL) was added into an EP tube and mixed vigorously for 30 s, and then allowed to sit for another 5 min at RT. Next, the lysate was centrifuged for 10 min 4 °C at 12,000×*g*, and the liquid in the centrifuge tube produced different layers after centrifugation. The supernatant was carefully pipetted to a new 1.5 mL EP with a small pipette. Isopropanol (0.5 mL) was added and the mixture vigorously for 30 s, and allowed to sit for another 10 min at RT. Then, lysate was centrifuged again for 10 min 4 °C 12,000×*g*, leaving a precipitate at the bottom of the tube. Next, 75% ethanol (1 mL, precooled at 4 °C) was added and the precipitate was suspended again. After that, the EP was centrifuged for 15 min 4 °C at 12,000×*g*, and the supernatant was discarded. An appropriate amount of diethylpyrocarbonate (DEPC) H_2_O (and incubated at 65 °C to promote dissolution) was added based on the size of the precipitation when the EP tube was air drying. The tube was oscillated to dissolve the precipitate. The concentration of total RNA was measured by Nanodrop.

### Total antioxidant capacity (TAOC) assay

TAOC of the sera was determined colorimetrically at 570 nm according to the instructions of a commercial TAOC assay kit (Abcam).

### ELISA

The levels of IL-1β in the blood serum were determined using a commercial ELISA kit (Abcam) according to the manufacturers’ protocol.

### mRNA sequencing

A total amount of 1 µg RNA per sample was used as input material for the RNA sample preparations. Sequencing libraries were generated using NEBNext^®^ Ultra™ RNA Library Prep Kit for Illumina^®^ (NEB, USA) following the manufacturer’s recommendations and index codes were added to attribute sequences to each sample.

The clustering of the index-coded samples was performed on a cBot Cluster Generation System using TruSeq PE Cluster Kit v3-cBot-HS (Illumina) according to the manufacturer’s instructions. After cluster generation, the library preparations were sequenced on an Illumina Novaseq platform and 150 bp paired-end reads were generated.

### Sequencing data analysis

Raw data (raw reads) of fastq format were first processed using in-house perl scripts. In this step, clean data (clean reads) were obtained by removing reads containing adapter sequences, reads containing ploy-N regions, and low-quality reads from raw data. At the same time, Q20, Q30, and GC content of the clean data were calculated. All the downstream analyses were based on clean data with high quality.

The reference genome and gene model annotation files were downloaded from the genome website directly. Index of the reference genome was built using Hisat2 v2.0.5 and paired-end clean reads were aligned to the reference genome using Hisat2 v2.0.5.

Differential expression analysis of the two conditions (two biological replicates per condition) was performed using the DESeq2 R package (1.16.1). DESeq2 provides statistical routines for determining differential expression in digital gene expression data using a model based on the negative binomial distribution. The resulting P-values were adjusted using the Benjamin and Hochberg’s approach to control the false discovery rate. Genes with an adjusted P-value < 0.05 defined as DESeq2, were assigned as differentially expressed.

### GO and KEGG enrichment analysis of differentially expressed genes

Gene Ontology (GO) enrichment analysis of differentially expressed genes was implemented by the cluster profile R package, in which gene length bias was corrected. GO terms with a corrected P-value < 0.05 were considered significantly enriched by differentially expressed genes.

Kyoto Encyclopedia of Genes and Genomes (KEGG) is a database resource for understanding high-level functions and utilities of biological systems, such as the cell, the organism, and the ecosystem, using molecular-level information, especially using large-scale molecular datasets generated by genome sequencing and other high-throughput experimental technologies (http://www.genome.jp/kegg/). We used a cluster profile R package to test the statistical enrichment of differential expression genes in the KEGG pathways.

### Quantitative polymerase chain reaction

Total RNA was reverse transcribed to cDNA using a Reverse Transcription Kit (Takara, Dalian, China). Real-time PCR analyses (qPCR) were performed with SYBR Green (Takara, Dalian, China) and the following amplification parameters were used: 95 °C for 1 min, 95 °C for 10 s, and 60 °C for 60 s (40 cycles). The amplification mixture included 50 ng cDNA, 2 mM Mg^2+^, 100 pmol of each primer, 200 mM dNTP, and 2.5 μg Tag DNA polymerase. Results were normalized to the expression of glyceraldehyde-3-phosphate dehydrogenase (GAPDH) and the relative expression of genes was calculated using the 2^−ΔΔCt^ method. The primers used are listed in Table 1. The ABI 7500 fast real-time PCR system (ABI, USA) was employed for the PCR analyses.

**Table 1:** The sequence of the primers used for qRT-PCR in this research

Gene	Primers sequence
HIF-1α	Forward: 5′-CATCGAGCCACTCTTTGACC-3′
	Reverse: 5′-GCCATTAGGTAGGTGTAGCTGG-3′
VEGF	Forward: 5′-CCTGGCAAAGAGAAGACACAGTG-3′
	Reverse: 5′-GAAGGGTAAGCCACTCACACAC-3′
CGB	Forward: 5′-CATTCTTCCAGACCAGGG-3′
	Reverse: 5′-CATCTTTGGTGTTGCCTGAG-3′
EPO	Forward: 5′-CTCTTCCGGGTCTACTCCAAC-3′
	Reverse: 5′-GGTTCTCTCAGTCTCTCCTGCTC-3′
MMP8	Forward: 5′-CAATGGAATCCTTGCCCATG-3′
	Reverse: 5′-GTGCTGGGTTCTCTGTAAGC-3′
MMP9	Forward: 5′-CCCTTGAACTAAGGCTCCTC-3′
	Reverse: 5′-GACAAGCTGATTGGTTCGAG-3′
IL-17b	Forward: 5′-CTGAGGAACAGCTCTGAGCC-3′
	Reverse: 5′-CTCACCATGCTACGGTCCTC-3′
TIMP1	Forward: 5′-GAACCGCAGCGAGGAGTTTC-3′
	Reverse: 5′-CAAGCAATGACTGTCACTCTCC-3′
Ccl2	Forward: 5′-GCAGCAGGTGTCCCAAAGAAG-3′
	Reverse: 5′-GTGGAAAAGAGAGTGGATGC-3′
Ccl7	Forward: 5′-GAAGACAGATGCCTGAACAG-3′
	Reverse: 5′-GTGACATGAGGTCTCCAGG-3′
TNFSF9	Forward: 5′-GAGAATACTGCAGACCAGGTC-3′
	Reverse: 5′-GACTGTCCACCACCAACTC-3′
FAIM	Forward: 5′-GGAGCTGCAAAGACCAAAG-3′
	Reverse: 5′-CAGTCCCGTCATCTACAAAC-3′
S100a8	Forward: 5′-GGCAACTGAACTGGAGAAGG-3′
	Reverse: 5′-CCCTTATCACCAACACAAGG-3′
S100a9	Forward: 5′-CATCCTGACACCCTGAACAAG-3′
	Reverse: 5′-GTGGGTTGTTCTCATGCAG-3′

### Western blot analysis

The tissues were fully lysed and sonicated, and the total protein was extracted. Protein levels were quantified using a BCA Protein Assay Kit. Polyacrylamide gel electrophoresis was performed with 10 μg protein from each sample. Next, proteins on the gel were transferred onto a polyvinylidene fluoride (PVDF) membrane by semi-dry transfer, and the PVDF membrane was sealed with 5% skim milk powder for 2 h at RT. Thereafter, the membrane was probed with the primary antibody at 1:1000 dilution with phosphate buffer saline (PBS) against matrix metalloproteinase 8 (MMP8), MMP9, IL-17β, tissue inhibitor of metalloproteinase 1 (TIMP1), Ccl2, Ccl7, TNF ligand superfamily member 9 (TNFSF9), and Fas apoptotic inhibitory molecule (FAIM), S100a8, S100a9 at 4 °C overnight. All primary antibodies were provided by Cell Signaling. The PVDF membrane was then washed three times with PBS-Tween for 8 min each. The secondary antibody was diluted with PBS and was incubated for 1 h at RT, and the PVDF membrane was washed again. β-actin was used as a loading control. Proteins were visualized using an enhanced chemiluminescence solution. The following antibodies were used: MMP8 (Abcam, ab81286), MMP9 (CST, 13667T), IL-17β (Abcam, ab125029), TIMP1 (CST, 8946S), Ccl2 (Abcam, ab186421), Ccl7 (Abcam, ab182793), TNFSF9 (Abcam, ab68185), FAIM (Abcam, ab154570), S100a8 (CST, 47310T), and S100a9 (CST, 73425S).

### Statistical analysis

Statistical analysis was performed using PASW Statistics for Windows, Version 18.0 (SPSS Inc., Chicago, IL, USA) and GraphPad Prism Software (GraphPad Software, Inc., San Diego, CA, USA). Comparisons between the three groups were analyzed with one-way Analysis of Variance (ANOVA), and the multiple comparisons were analyzed with Tukey. A P-value < 0.05 was considered to indicate significant differences. Each experiment was performed at least three times, and the results were expressed as the mean ± standard deviation (SD).

## Results

### QLT1 alleviated lung injury and immune cell infiltration in a rat AHH model

**Fig. 1:**
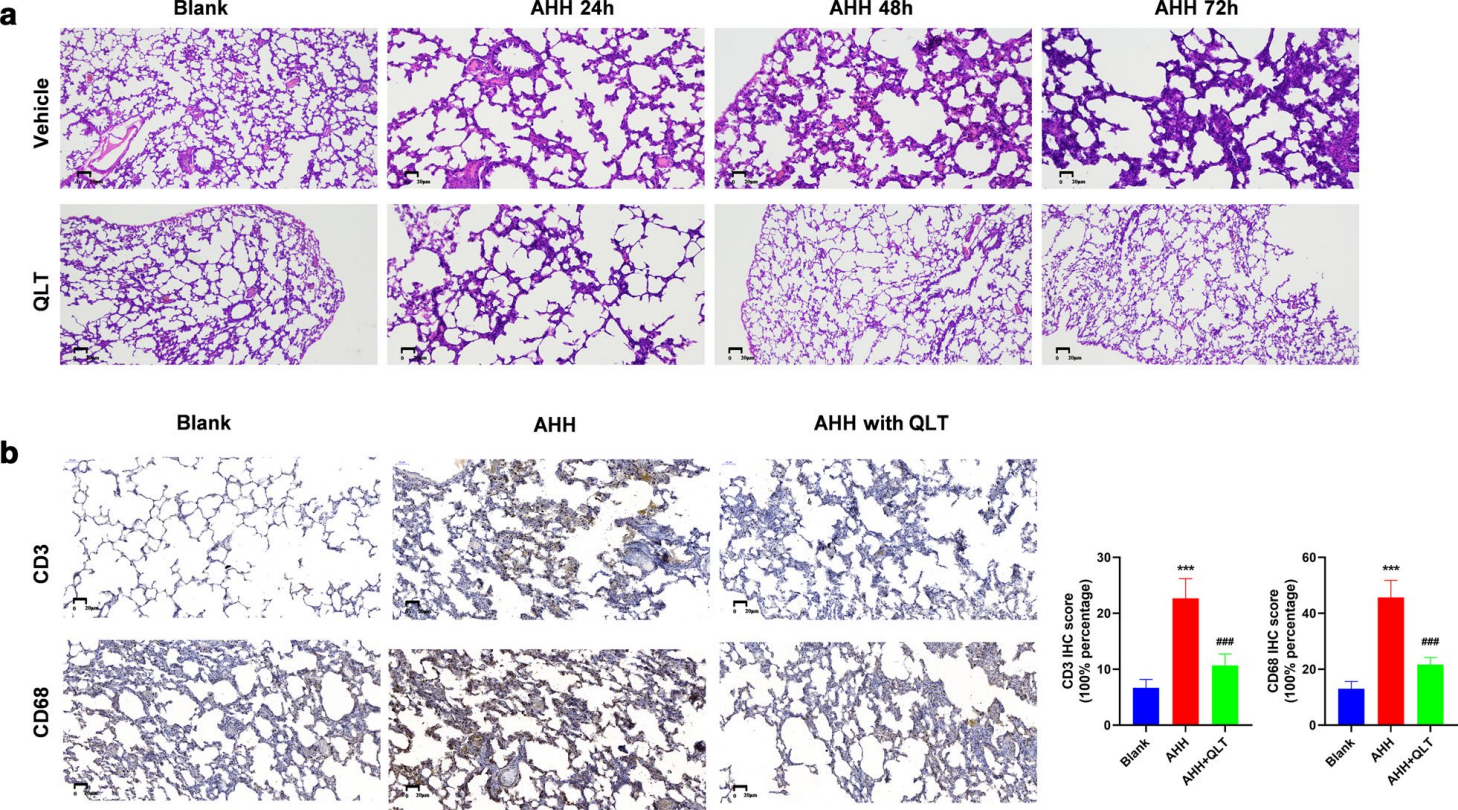
Phenotype of rat lung after the rats were exposed to AHH or AHH and QLT treatment. **a** HE staining for pathological changes of rat lung of each group. **b** IHC staining of CD3 and CD68 of the rat lung of each groups after a 72-h exposure. ***P < 0.001, *blank vs. AHH, ^#^AHH vs. QLT + AHH

We used hematoxylin–eosin staining to observe the damage to the lung tissue of rats under AHH conditions. The lung tissue of the blank group showed no obvious pathological changes, and the alveoli were regular and intact. There was no obvious edema of the alveolar interval nor immune cell infiltration. However, the alveolar septa thickened under AHH for 24 h and presented immune cell infiltration and capillary congestion. After 48 h the alveolar septa of the rats in AHH were broken; the lung tissue structure was destroyed at 72 h, in addition changes in the alveolar septa, and the capillaries were dilated and a presented a large number of infiltrated inflammatory cells. QLT treatment significantly alleviated the structural changes at 72 h under AHH (Fig. 1a).

In lung tissues under AHH conditions, alveoli walls presented immune cell infiltrated. IHC staining of CD3 and CD68 were employed to assess T cell and macrophage cell infiltration, and to compare the level of inflammation between the AHH and QLT + AHH groups at the 72-h time point. T cells and macrophage cells were significantly infiltrated in the alveoli walls. While the QLT treatment significantly alleviated the degree of immune cell infiltration (Fig. 1b).

### QLT1 alleviated TAOC and hypoxia induces inflammation in rat AHH model

**Fig. 2:**
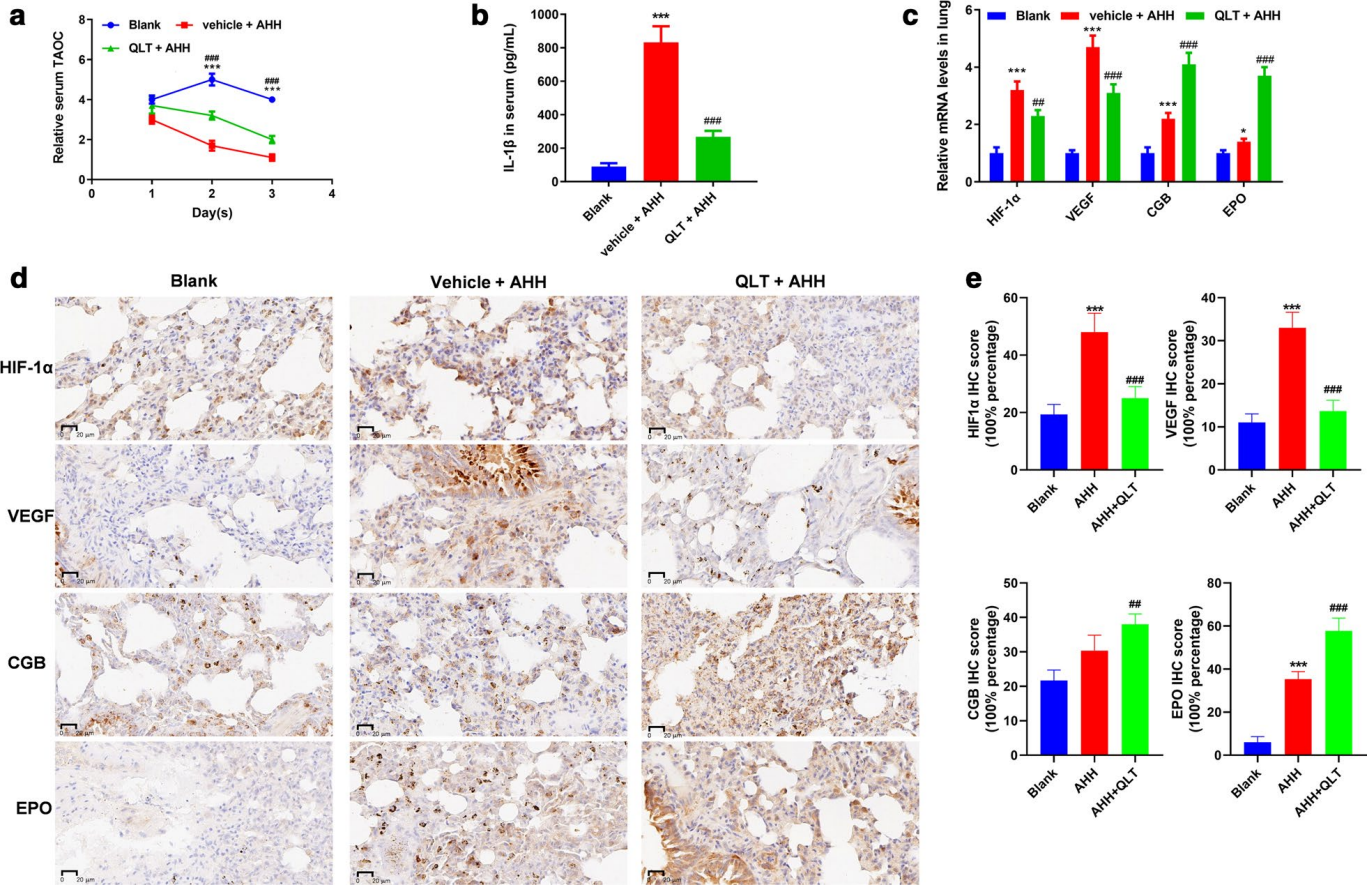
The evaluation of biochemical characteristics of the rat model exposed to AHH vs. QLT + AHH for 72 h. **a** The relative levels of TAOC in the sera of rats. **b** Concentrations of IL-1β in the sera of rats. **c** Relative mRNA levels in the lung tissues of rats. **d** Levels of the indicated proteins in the lung tissues of rats. **e** IHC score of each protein stained with IHC. **P < 0.01, ***P < 0.001; *blank vs. AHH, ^#^AHH vs. QLT + AHH

TAOC reflects the capacity of the body's overall defense against AHH-induced oxidative damage. TAOC significantly decreased at 48 h post-AHH, and in the AHH group was one-third that of the blank group (P < 0.05). TAOC levels decreased to the lowest level at the 72-h time point. QLT treatment partially resorted the TAOC levels under AHH (Fig. 2a).

Inflammation is an important factor for promoting acute high-altitude diseases. Hypoxia induces inflammation and the release of inflammatory factors. AHH dramatically increased the level of IL-1β compared with the blank condition, at the 72-h time point, although QLT suppressed the effect of AHH by maintaining the concentration of IL-1β close to the basal level (Fig. 2b). HIF-1α is highly sensitive to oxygen levels and defines “the switch of hypoxia-related gene expression”. In this model, AHH stimulated HIF-1α expression, which was partially reversed by QLT treatment. In HAPE, high VEGF expression contributes increase pulmonary vascular permeability. AHH caused an increased expression of VEGF, but QLT treatment significantly inhibited VEGF levels in AHH lung tissues. Cytoglobin (CGB) is a hypoxic protective factor with oxygen-carrying capacity and protection against oxidative stress; erythropoietin (EPO) exerts anti-oxidative stress effects and steroid hormone-like anti-inflammatory effects. Expression of both CGB and EPO were stimulated under AHH as a response to low PO_2_ conditions, and QLT further induced their levels to alleviate damage to the lung (Fig. 2c–e).

### RNAseq analysis of the differential expressed genes

**Fig. 3:**
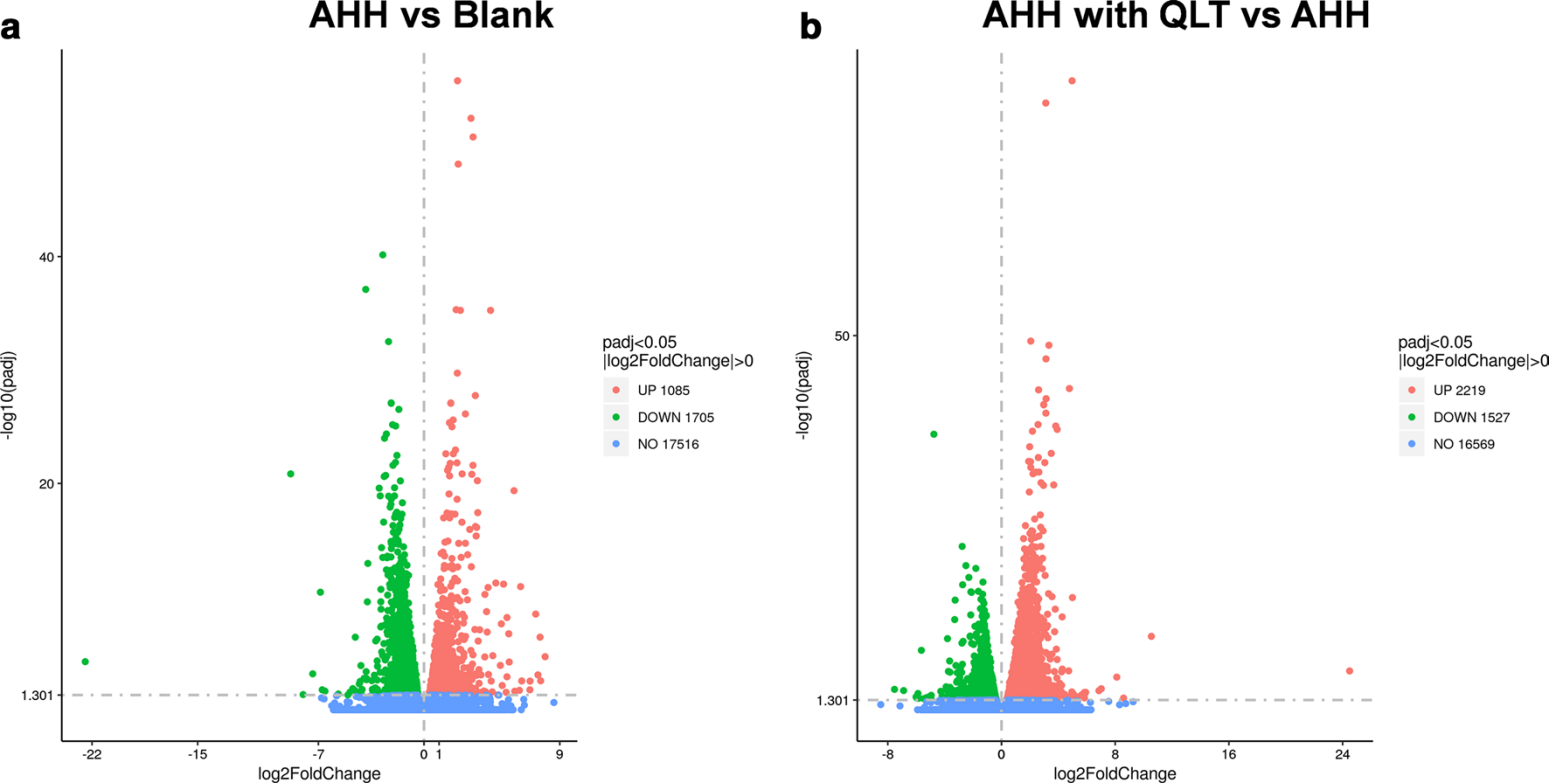
Volcano plots showed differentially expressed genes in the lung tissues of the rats after exposure to AHH for 72 h

Volcano charts were plotted based on two factors, fold-change and the p-values obtained by t-test, show the significant differences in data between the two groups of samples (Fig. 3). The results showed that there were 1085 up-regulated genes compared with the blank compared to the AHH conditions, and 1705 down-regulated genes, predicted to be involved in the occurrence of acute high-altitude diseases. The comparison of QLT- and saline-treated AHH rat showed that QLT induced the expression of 2219 genes, and down-regulated the expression of 1527 genes, which may mediate the actions of QLT in vivo.

**Fig. 4:**
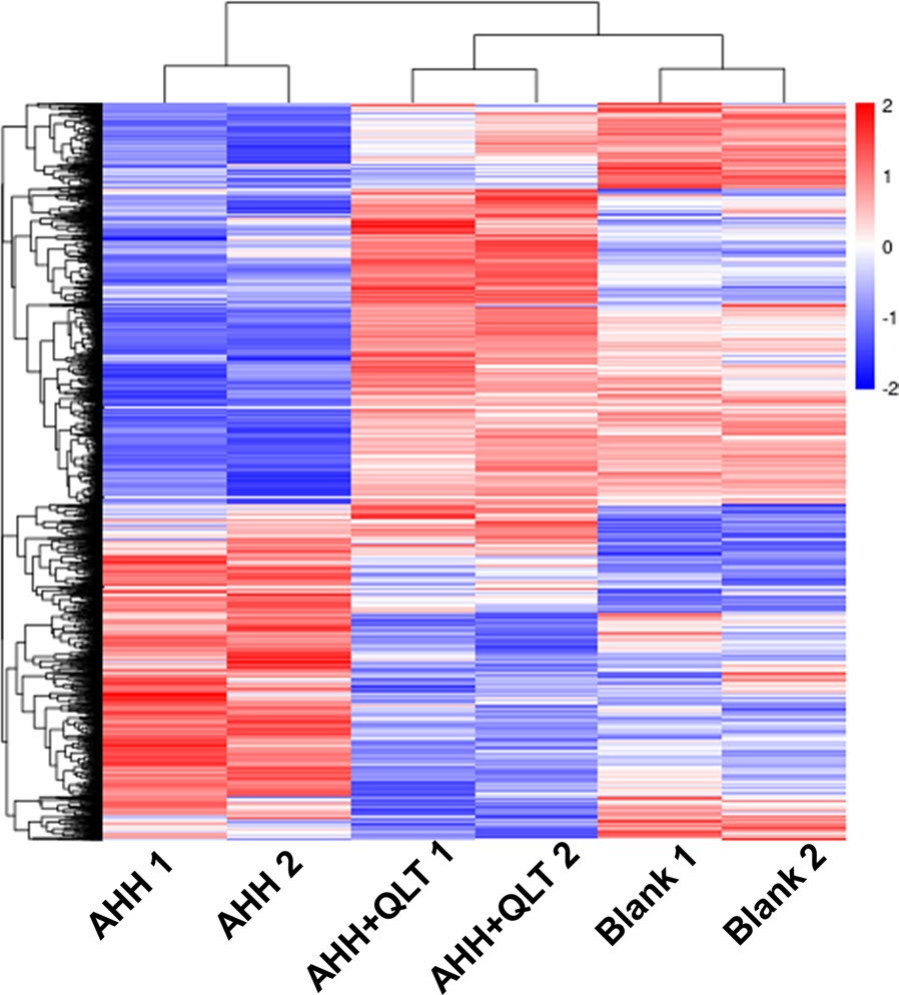
Hierarchical clustering of differentially expressed genes. For hierarchical clustering, blue and red indicate decreased and increased expression, respectively. The proteins were clustered by hierarchical clustering using the complete linkage algorithm and Pearson correlation coefficients in R

We used hierarchical clustering to compare differentially expressed genes of representative cases as shown in the heat map (Fig. 4). The chart indicated that the threshold fold-change set in the current study can effectively separate the blank, AHH, and QLT-treated AHH rats, and the data of each case from the three groups were reproducible.

### GO and KEGG analysis of the differential signaling pathway

**Fig. 5:**
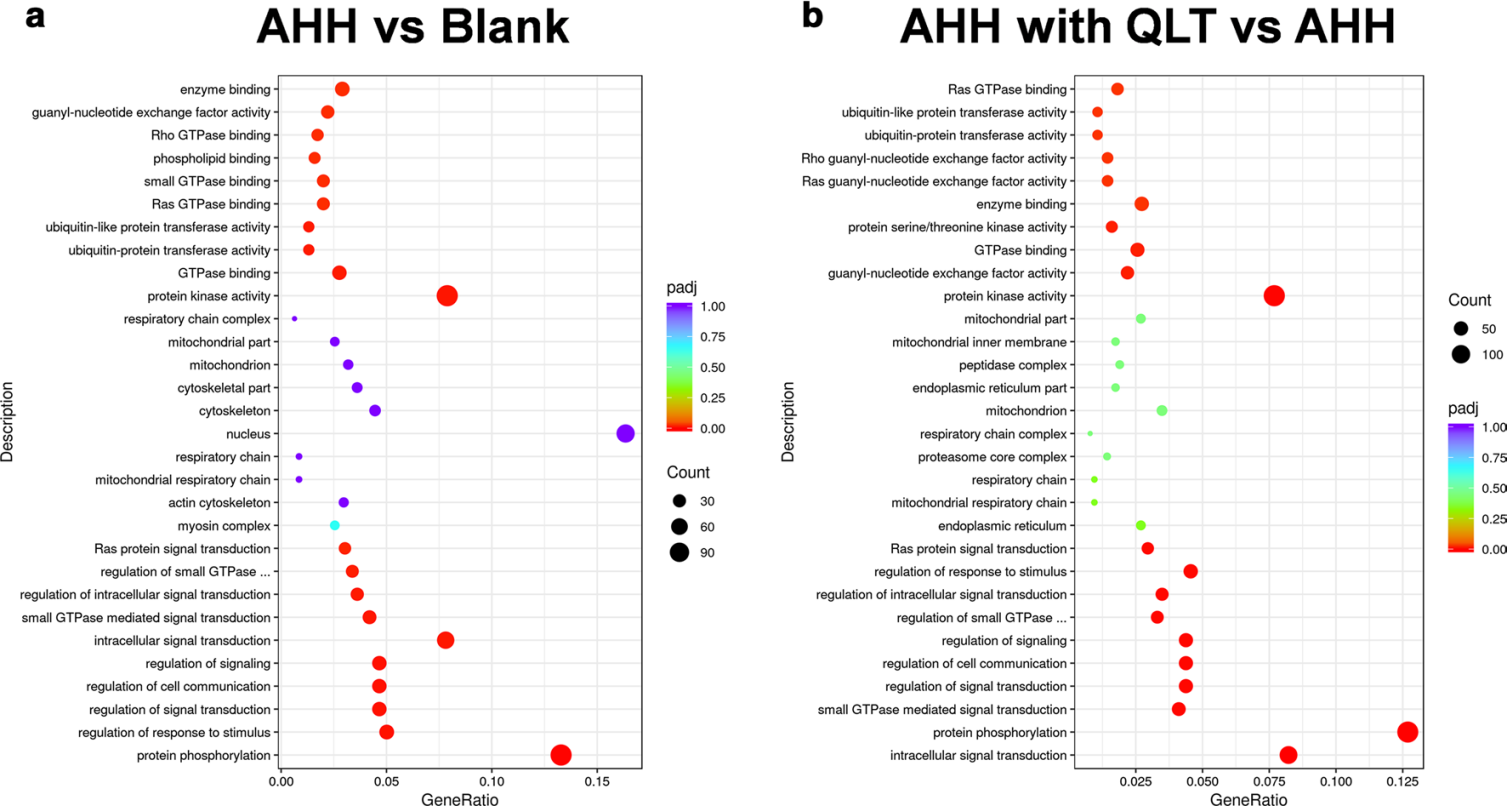
GO classification of differentially expressed proteins by biological process, cellular component, and molecular function

The differentially expressed genes were annotated according to the biological processes, cellular components, and molecular functions involved by BLAST2TO (Fig. 5). The major biological processes of the differentially expressed genes in the comparison between the blank and AHH rats were protein phosphorylation, regulation of response to stimuli, and regulation of signal transduction. The cellular component analysis showed that most were located in the nucleus, cytoskeleton, and mitochondria. The differentially expressed genes mainly play a role in protein kinase activity, GTPase binding, and ubiquitin-protein transferase activity. In the comparison of QLT- and saline-treated AHH rats, QLT activities may include the biological processes of intracellular signal transduction, protein phosphorylation, and small GTPase mediated signal transduction. The differentially expressed genes following QLT treatment were mainly associated with the endoplasmic reticulum, mitochondrial respiratory chain, and proteasome core complex. These genes responsive to QLT treatment under AHH, involved the molecular functions of protein kinase activity, GTPase binding, and protein serine/threonine kinase activity.

**Fig. 6:**
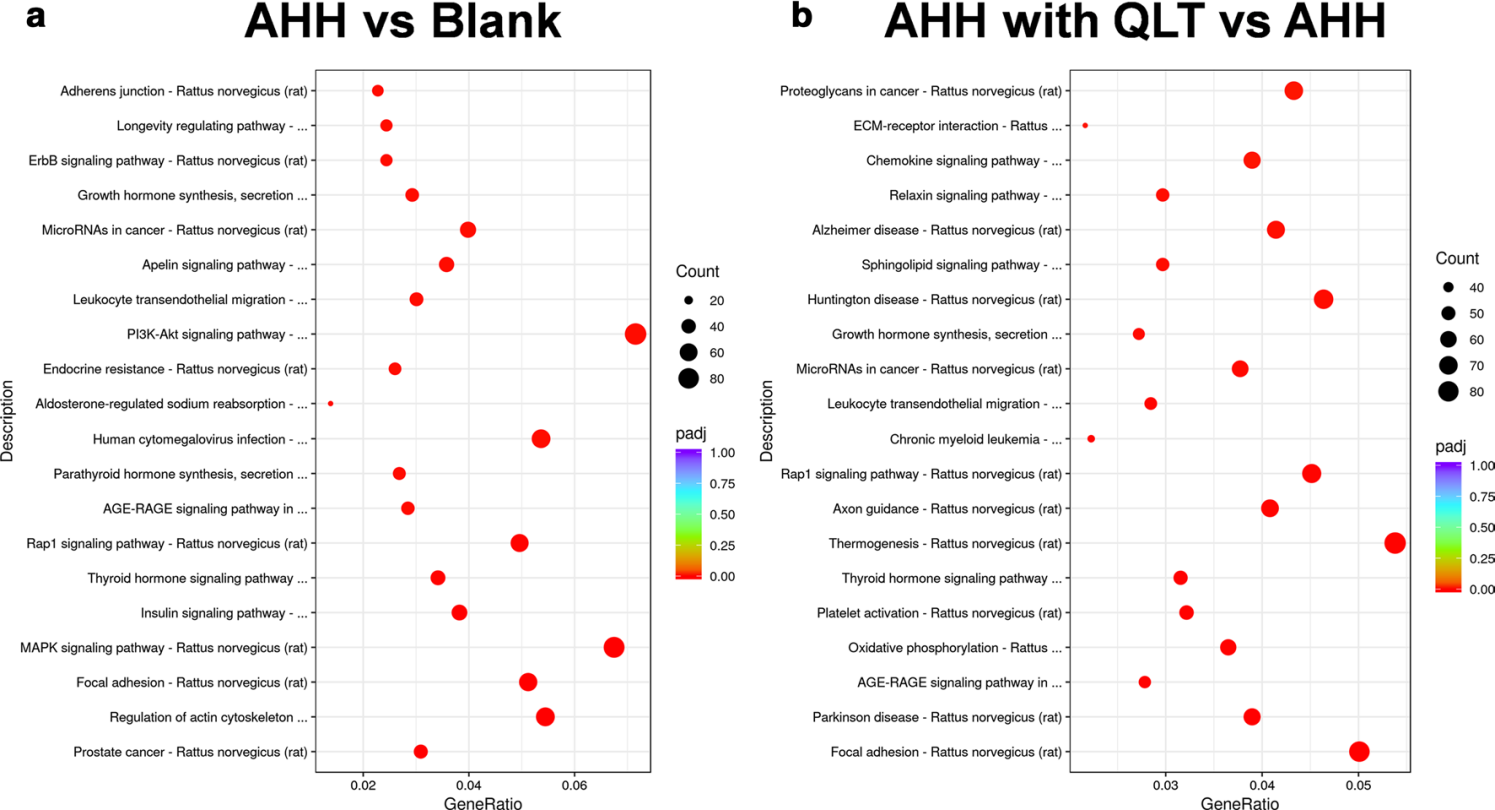
KEGG analysis of the differentially expressed proteins. To the left of each plot: KEGG terms. Under each plot: the percentage of the sequence

Pathway annotation by KEGG analysis demonstrated that the occurrence of acute high-altitude diseases was associated with the regulation of the actin cytoskeleton, MAPK signaling pathway, and focal adhesion. Moreover, QLT may function in AHH rats by modulating focal adhesion, the AGE-RAGE signaling pathway, and oxidative phosphorylation (Fig. 6). From the bioinformatics analyses, we can begin to postulate the mechanisms involved in AHH-induced pulmonary damage and the protective effects induced by QLT at the molecular level.

### Validation of the RNAseq results and mechanism of QLT alleviate inflammatory response

**Fig. 7:**
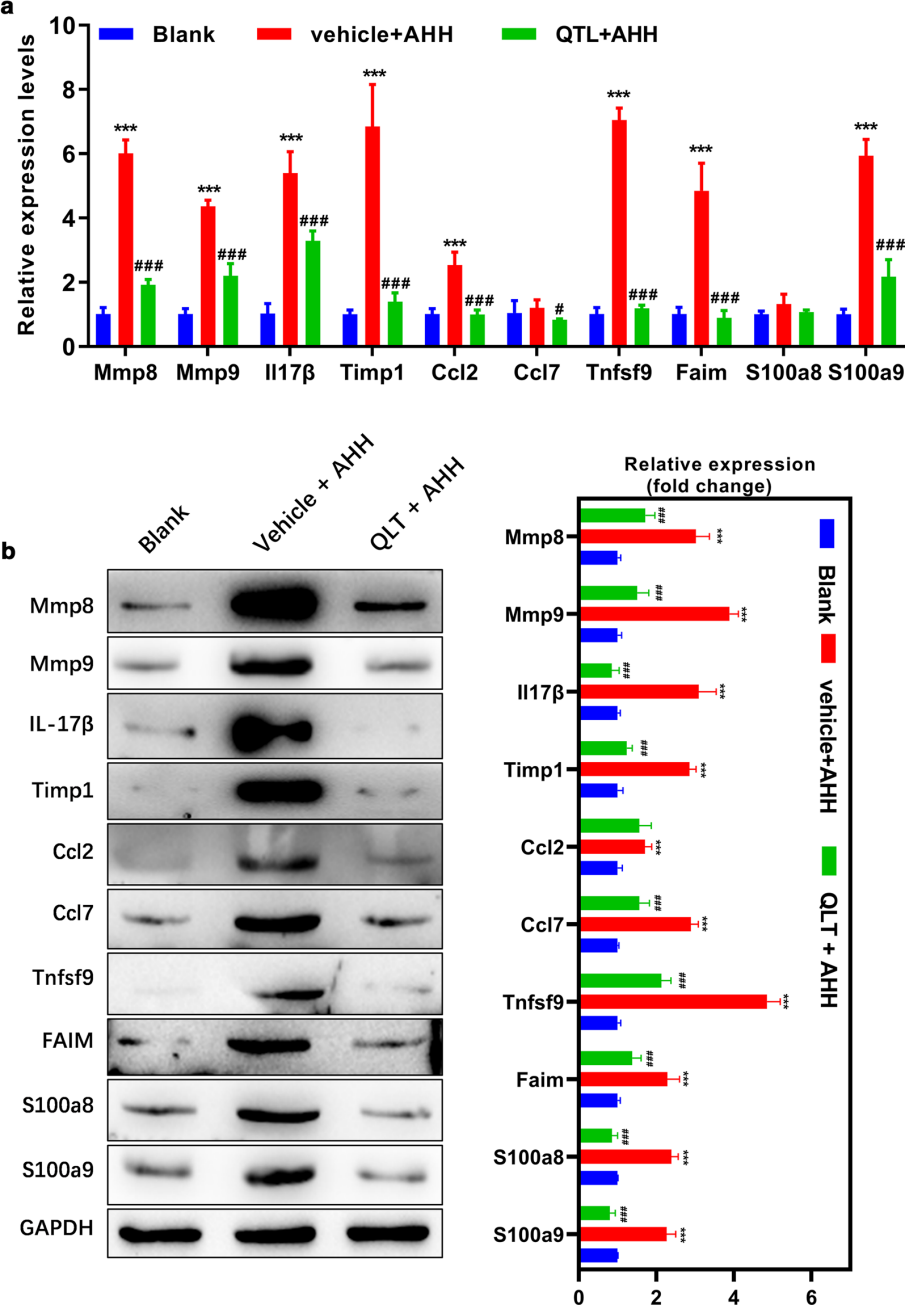
Relative expressions of the inflammatory markers at the mRNA (**a**) and protein (**b**) levels. **P < 0.01, ***P < 0.001; *blank vs. AHH, ^#^AHH vs. QLT + AHH

By reviewing the list of the top 200 differentially expressed genes, we found that many inflammation-related genes such as MMP8, MMP9, IL-17β, and Timp1 were significantly upregulated in the rats exposed to AHH, whose expression was repressed by QLT administration. Thus, we deduced that QLT might function via suppressing the inflammation brought by hypobaric hypoxia. Next, we verified the changes in mRNA and protein levels in lung tissues. As shown in Fig. 7, the trend in expression of these molecules was consistent with the sequencing data, though the differences in expression of Ccl7 and S100a8 were not significant at the mRNA level. Herein, we may conclude that QLT alleviated the inflammatory responses under AHH conditions in the rat model, which could be responsible for relieving symptoms of acute high-altitude diseases.

## Discussion

TCM has shown to be effective and safe for the treatment of acute high-altitude diseases. QLT, a formula for “BuQi”, “HuoXue”, “TongLuo”, and “LiShui” according to the theory of TCM, is composed of 3 selected medicinal materials prepared following the traditional criteria. However, as a TCM formula, QLT presents difficulties in the standardization of its composition and in clarifying the mechanisms of action involving the multi-component composition [20]. In the present study, we investigated the molecular mechanisms involved in QLT effects on alleviating the symptoms of acute high-altitude diseases. Using a rat model treated under AHH, we evaluated the structural alterations in lung tissues using HE staining. Other biochemical indexes like TAOC, IL-1β, and HIF-1α also indicated that the animal models were injured following the simulated hypobaric hypoxia environment. The mRNA-Seq analyses revealed that multiple pathways were involved in the damage induced by AHH and the protective mechanisms activated by QLT treatment. KEGG enrichment indicated that the differentially expressed genes mainly belonged to the PI3K–AKT, focal adhesion, MAPK, and Rap1 signaling pathways. By reviewing the list of altered genes, we found many inflammatory molecules ranked high according to the fold-changes induced under AHH conditions. Moreover, genes were also sensitive to QLT treatment by showing a significantly reversed expression. Therefore, it is reasonable to deduce that QLT relieves the symptoms of acute high-altitude diseases by inhibiting the inflammatory responses induced by AHH.

We found a large proportion of differentially expressed genes derived from phosphorylation-related pathways, which were closely associated with hypoxia stress. Previous studies [21] have confirmed that hypoxia can stimulate the PI3K/AKT pathway by phosphorylating AKT, which induces the transcription of HIF-1α and stabilizes HIF-1α protein, which subsequently increases the expression of EPO and VEGF to promote erythropoiesis and angiogenesis. Hypoxia results in a significant increase in reactive oxygen/nitrogen species, which act as signaling molecules for activating the MAPK pathway. Downstream JNK, ERKs, and p38 were sequentially activated by phosphorylation in response to hypoxia. Next, the MAPK pathway up-regulated the downstream targets, phosphorylated c-Jun and JunB, causing inhomogeneous vasoconstriction leading to vascular leakage and inflammation over longer exposures to hypobaric hypoxia [22]. Therefore, the findings from this study also indicated that we should pay greater attention to protein phosphorylation in rat models in the future.

In addition, we also verified the responses of inflammatory-associated proteins to QLT treatment, and they were among the most significantly differentially expressed molecules. These molecules may be effectors of QLT in this model active in relieving the symptoms of acute altitude sickness. HIF-1α induces iNOS expression, which increased levels of nitric oxide and activated MMPs in various cells and tissues [23], thereby stimulating the activity of MMP9. This protease promotes the degradation of extracellular components and vascular basement membrane. Hypoxia-induced cell invasion was significantly inhibited by specifically silencing the HIF-1α/MMP9 signaling pathway, indicating MMP9 plays a vital role [24]. Airway remodeling is caused by the dysregulation of MMPs and as a key regulator of the extracellular matrix, MMP9 participates in the degradation of various structural proteins including collagen and elastin. MMP9 activity in turn is affected by TIMP1, which acts as both an inhibitor and a substrate for MMP9 [25]. Our study shows that TIMP1 and MMP9 were both involved in the pathogenesis of high-altitude diseases. Ccl2, MMP9, S100a9, and S100a8 are downstream target genes of IL-17, which are involved in the pathogenesis of autoimmune responses, neutrophil recruitment, and immunity to extracellular pathogens [26]. Hypoxic conditions may enhance the permeability of pulmonary blood vessels, which in turn promote alveolar macrophages to release Ccl2 to recruit inflammatory monocytes, and at the same time upregulate S100a8 and S100a9 [27].

## Conclusion

Herein, we analyzed the potential molecular mechanisms underlying acute high-altitude diseases such as AMS, which share similarities with other pulmonary disorders. We identified the inflammation-related effectors activated by QLT treatment against AHH, and described several phosphorylation signaling pathways involved in the pathogenesis of the disease. Although we believe that additional studies are needed to further clarify the underlying mechanisms, especially those involving protein phosphorylation, the present study provides insights relative to the major signaling pathways of AHH and the protective effects of the QLT formula.

## Data Availability

All data generated or analyzed during this study are included in this manuscript.

## Abbreviations

PO_2_: The pressure of oxygen
AMS: Acute mountain sickness
AHH: Acute hypobaric hypoxia
HAPE: High-altitude pulmonary edema
TCM: Traditional Chinese medicine
QLT: Qi-Long-Tian
HE: Hematoxylin–eosin
RT: Room temperature
DEPC: Diethyl pyrocarbonate
TAOC: Total antioxidant capacity
GO: Gene Ontology
KEGG: Kyoto Encyclopedia of Genes and Genomes
qPCR: Quantitative polymerase chain reaction
GAPDH: Glyceraldehyde-3-phosphate dehydrogenase
PVDF: Polyvinylidene fluoride
PBS: Phosphate buffer saline
TNFSF9: Tumor necrosis factor ligand superfamily member 9
FAIM: Fas apoptotic inhibitory molecule
SD: Standard deviation
